# Management of Extramammary Paget Disease of the Male Genital Region: A Narrative Review and Two Case Reports

**DOI:** 10.3390/jcm15041355

**Published:** 2026-02-09

**Authors:** Marta Labon, Katarzyna Czajkowska, Marcin Matuszewski, Mateusz Czajkowski

**Affiliations:** 1 Student Research Group, Department of Urology, Medical University of Gdańsk, 80-210 Gdańsk, Poland; 2 Department of Dermatology, Medical University of Gdańsk, 80-210 Gdańsk, Poland; k.zalewska@gumed.edu.pl; 3 Department of Urology, Medical University of Gdańsk, 80-210 Gdańsk, Poland

**Keywords:** extramammary Paget disease, penoscrotal extramammary Paget disease, photodynamic therapy, positron emission tomography–computed tomography

## Abstract

**Background/Objectives**: Extramammary Paget disease (EMPD) is a rare, slow-growing intraepithelial malignancy arising in apocrine gland-bearing skin, most commonly in the anogenital region. Although often confined to the epidermis, EMPD may be associated with synchronous or underlying malignancies and can progress to invasive disease, making early recognition and compre- hensive staging crucial. **Methods**: We review current knowledge on the epidemiology, molecular pathogenesis, diagnostic work-up and treatment of EMPD with a particular focus on male genital involvement, and illustrate key clinical issues with two cases of penoscrotal EMPD treated in our center. Clinically, EMPD typically presents as chronic erythematous, pruritic plaques that are frequently misdiagnosed as benign dermatoses, leading to diagnostic delays. **Results**: Histopathology with immunohistochemistry remains the diagnostic gold standard and guides the search for associated internal malignancies. Wide local excision and Mohs micrographic surgery are the mainstays of treatment, but recurrence is common owing to subclinical extension. Non-surgical modalities—including photodynamic therapy, topical imiquimod, radiotherapy and, in advanced disease, systemic chemotherapy, HER2 targeted agents and immune checkpoint inhibitors—provide additional options in selected patients. **Conclusions**: A multidisciplinary, biomarker driven approach is essential to individualize management and improve long-term outcomes in this challenging disease.

## 1. Introduction

EMPD is a rare, slow-growing cutaneous adenocarcinoma that predominantly affects areas of the body with a high concentration of apocrine glands. Typical sites include the genital and perianal regions, penoscrotal skin, groin and inner thighs [1,2]. The disease may present as carcinoma in situ confined to the epidermis or as invasive disease with potential metastases to regional lymph nodes and distant organs. In male patients, the penoscrotal area is the most common site of both in situ and invasive EMPD [3]. EMPD exhibits distinct clinical and epidemiological patterns across different populations (Table 1). In large series, the most frequently affected site is the vulva, accounting for approximately 65 percent of cases, whereas perianal disease represents about 20 percent [3,4]. In Caucasians, EMPD occurs predominantly in women, with male-to-female ratios ranging from 1:2 to 1:7 [3,5]. By contrast, in Asian populations EMPD more often affects men, with preferential involvement of the scrotum, penis and groin, and male-to-female ratios between 2:1 and 4:1 [3,5]. Across populations, EMPD is mainly a disease of older adults, with typical age at diagnosis between 60 and 80 years, and a median diagnostic delay of approximately 2 years [5,6,7]. An important clinical feature of EMPD is its association with underlying or synchronous malignancies, reported in 9–32 percent of patients [6,8,9]. The most frequent associated cancers are colorectal adenocarcinoma and urothelial carcinoma of the bladder and urinary tract, followed by malignancies of the prostate, breast, cervix and endometrium [8,10,11]. The anatomical site of EMPD often correlates with the location of an associated carcinoma: perianal EMPD is most commonly linked to colorectal or anorectal cancer [2,12], whereas penoscrotal and inguinal EMPD are more frequently associated with urothelial carcinoma of the bladder or urinary tract and, less commonly, with prostate cancer [2,13]. This pattern underscores the need for comprehensive diagnostic evaluation, including detailed history and physical examination, cross-sectional imaging (with PET–CT where indicated) and site-specific screening such as colonoscopy or urological assessment depending on lesion location. Immunohistochemistry (IHC) plays a key role in distinguishing primary from secondary EMPD. Expression of GCDFP 15 and GATA3 favors a primary apocrine origin, whereas positivity for CK20 and CDX2 suggests secondary EMPD of colorectal origin; urothelial carcinomas typically express CK7 and GATA3 [5,14]. However, IHC findings must always be integrated with clinical and imaging data, as no single marker is sufficient for definitive classification. In this context, we present two cases of penoscrotal EMPD in male patients and discuss current concepts in the pathogenesis, diagnostic work-up and management of this rare malignancy. This review focuses specifically on extramammary Paget disease involving the male genital region, including the penis, scrotum, penoscrotal junction, and adjacent inguinal skin. The literature search prioritized studies and case reports describing EMPD of the male genital region. Articles focusing exclusively on female genital EMPD or non-genital EMPD were excluded unless they provided data relevant to diagnostic strategies, imaging, or treatment approaches potentially applicable to male genital EMPD. We included these two patients in our study because they exemplify the diagnostic delay and management complexity typical for this condition, while also demonstrating clinically relevant heterogeneity in presentation and/or treatment course. Together, the cases highlight key decision points, pitfalls in early recognition, and pragmatic aspects of care that may be generalizable to similar clinical settings.

## 2. Search Strategy and Selection Criteria

We performed a narrative literature review using PubMed/MEDLINE and Embase up to August 2025. The search strategy was based on Boolean logic, combining the core term “extramammary Paget disease” with anatomical site-related terms (e.g., male genital region, penoscrotal, scrotum, penis) and management-related terms (e.g., diagnosis, treatment, surgery, photodynamic therapy, PET–CT, reflectance confocal microscopy) using the operator “AND”. Alternative anatomical locations and treatment modalities were combined using “OR”. We prioritized original studies, larger case series, and recent reviews published in English. Additional references were identified by manual screening of reference lists. Articles focusing exclusively on female genital EMPD or non-genital EMPD were excluded unless they provided data relevant to diagnostic strategies or management approaches applicable to male genital EMPD.

## 3. Pathogenesis and Molecular Landscape

The pathogenesis of EMPD is complex and remains the subject of ongoing debate. Three main hypotheses regarding the cell of origin have been proposed: malignant transformation of epidermal keratinocyte stem cells, derivation from apocrine gland epithelial cells [19], and development from Toker cells [20,21], which are benign clear epithelial cells located in the basal layer of the nipple epidermis. Immunohistochemical studies demonstrating CK7, GCDFP 15, CEA and HER2 expression in EMPD lesions support an adnexal gland phenotype and favor an apocrine origin in many cases [22,23]. Molecular investigations have revealed recurrent somatic mutations in PIK3CA, ERBB2 (HER2), RAS family genes and TP53, implicating canonical oncogenic signaling pathways such as PI3K/AKT/mTOR and MAPK in EMPD pathogenesis [24,25]. Importantly, HER2 amplification is observed in a substantial subset of cases [26]. However, genetic heterogeneity across anatomical sites suggests that EMPD may arise from a spectrum of potential progenitor cells, and current lineage tracing and molecular data are still insufficient to definitively confirm a single model of origin. In parallel, biomarker studies have identified androgen receptor (AR) expression, PD-L1 upregulation, and tumor mutational burden (TMB) as relevant [27,28,29]. AR expression is consistent with apocrine differentiation, as normal apocrine glands are known to express AR. While the prognostic relevance of AR has been debated due to methodological and cohort differences, higher AR expression (when assessed semi-quantitatively) has been linked to malignant progression and enrichment in invasive and metastatic/recurrent lesions, supporting its evaluation as both a prognostic biomarker and a potential therapeutic target in advanced disease. These markers may not only contribute to a better understanding of tumor histogenesis but may also serve as potential predictive biomarkers for response to immunotherapy and targeted therapies [5,30]. In EMPD, the immunohistochemical and molecular profile of tumor cells provides important insights into histogenesis by reflecting their glandular differentiation and possible origin from apocrine structures or underlying adnexal or visceral malignancies. Moreover, several markers implicated in EMPD are associated with key oncogenic pathways and the tumor immune microenvironment, including HER2 signaling and immune checkpoint regulation. This biological context explains why such markers may not only aid in elucidating tumor origin but also serve as potential predictive biomarkers for response to targeted therapies and immunotherapy. Thus, EMPD pathogenesis is best conceptualized as a multifactorial and molecularly heterogeneous process, which has direct implications for personalized management strategies (Table 2).

## 4. Diagnostic Work-Up

The clinical presentation of extramammary Paget’s disease typically consists of an asymmetric erythematous plaque with scaling of varying severity. In more advanced cases, erosions, crusts, papillary lesions, or nodules may develop within the plaque. The lesions are commonly accompanied by pruritus, which over time may lead to lichenification. Cutaneous manifestations of extramammary Paget’s disease are frequently misdiagnosed as eczematous lesions, leading to a delay in establishing the correct diagnosis. Dermoscopy often reveals milky-red areas, polymorphic or glomerular vessels, and white zones, which may aid in differentiating EMPD from benign dermatoses [34,35]. Reflectance confocal microscopy (RCM) is a valuable non-invasive tool for margin mapping and monitoring. Diagnostic features include dark cells one to two times larger than keratinocytes and target-shaped structures with a bright core and dark halo, located in the epidermis or at the dermoepidermal junction. Although epidermal disruption and increased vascularity may be present, they are not specific for EMPD. The observed growth pattern resembles pagetoid spread as seen in melanoma [36,37]. Histopathological examination remains the diagnostic gold standard. Paget cells are large, pale-staining cells with prominent nuclei and nucleoli, occurring singly or in clusters within the epidermis. Dermal invasion in extramammary Paget disease has been reported in approximately 15–40 % of cases, although the exact frequency varies across studies and may be influenced by sampling and diagnostic criteria. It is a key prognostic feature associated with increased risk of lymph node involvement and poorer outcomes. Immunohistochemistry (IHC) is essential for confirmation. Primary EMPD typically shows positivity for CK7 and GATA3 [31,38]. CK20 positivity suggests secondary involvement from colorectal or urothelial carcinoma [5,31]. Advanced imaging techniques complement histopathology in assessing disease extent and staging. PET–CT with 18F-FDG can identify metabolically active residual lesions, nodal metastases, or distant spread [39,40], although interpretation may be complicated by postoperative changes. Sentinel lymph node biopsy (SLNB) may detect occult nodal metastases in invasive cases and can influence management decisions, although its prognostic value has not been definitively established. Published series suggest that the overall incidence of sentinel lymph node (SLN) positivity in extramammary Paget disease is approximately 15–21%, although this varies substantially with depth of tumor invasion [41]. In systematic reviews of SLNB in EMPD, around 21.2% of patients had positive SLN, with positivity rates of approximately 15.6% in microinvasive lesions and 72.7% in deep invasive lesions. Larger retrospective cohorts also report SLN positivity around 15% in clinically node-negative, invasive EMPD. These data underscore the strong association between deeper dermal invasion and nodal metastasis, highlighting the potential role of SLNB in risk stratification and guiding further management. In the two cases presented, sentinel lymph node biopsy was not performed, as both patients were clinically node-negative and nodal status was assessed using imaging modalities rather than surgical nodal staging. Magnetic resonance imaging (MRI) may assist in evaluating local tumor extent and deep tissue invasion [42], providing complementary information to PET–CT. Together, these methods support accurate staging, surgical planning, and long-term surveillance of EMPD. The therapeutic management of EMPD is challenging due to the tumor’s frequent subclinical extension and high recurrence rates. Conventional wide local excision (WLE) remains a standard approach but is associated with recurrence rates ranging from 20 percent to 60 percent [43]. In contrast, Mohs micrographic surgery (MMS) or staged excision with complete circumferential peripheral and deep margin assessment has demonstrated significantly lower recurrence rates by ensuring meticulous margin control [44]. These methods are increasingly favored in centers with expertise, especially for recurrent or ill-defined lesions. Non-surgical approaches are gaining importance, particularly in patients unfit for surgery or with superficial disease. PDT, often combined with surgery or topical agents, has demonstrated efficacy in reducing recurrence and improving local control [45]. Nevertheless, the effectiveness of PDT declines in invasive lesions, highlighting its role as an adjunct rather than a replacement for surgery. Topical therapies, such as imiquimod and 5-fluorouracil (5-FU), have shown complete or partial responses but recurrence remains an issue and durability of response is limited [46]. For advanced or metastatic EMPD, systemic approaches are required. Traditional chemotherapy regimens (taxanes, platinum-based agents, 5-FU) provide transient responses with limited resilience [47,48]. With growing evidence of HER2 overexpression in a significant subset of extramammary Paget’s disease (EMPD), targeted therapies have transformed management in advanced and metastatic disease. In particular, trastuzumab, either as monotherapy or in combination with paclitaxel, has led to satisfactory clinical responses. Additionally, novel HER2-targeting agents or antibody–drug conjugates (ADCs)—disitamab vedotin—are under development and are showing promise in HER2-expressing malignancies, offering future potential in EMPD therapy [23,49]. Similarly, immune checkpoint inhibition has begun to demonstrate efficacy in select advanced EMPD cases. Notably, a case of EMPD with high TMB achieved partial lymph-node regression following pembrolizumab monotherapy [50]. Furthermore, radiotherapy remains an important modality—especially in palliative settings—for unresectable, recurrent, or metastatic EMPD. This method is reporting meaningful local disease control and symptom relief [51]. As a result, optimal treatment strategies for EMPD are increasingly multimodal and personalized, integrating surgical excision with non-surgical adjuncts, including systemic targeted therapies, immunotherapies and radiotherapy, tailored to disease stage and biomarker profile (Table 3).

## 5. Case Presentation I: Non-Invasive Penoscrotal EMPD Treated with Surgery and PET–CT Surveillance

**Figure 1:**
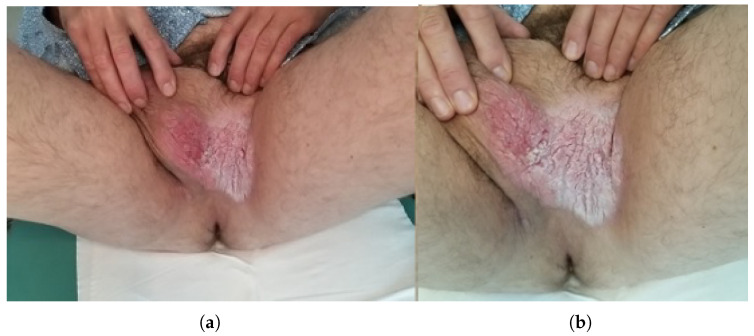
Preoperative clinical presentation of penoscrotal extramammary Paget disease. (**a**) Initial clinical appearance at presentation to the Urology Department, showing an erythematous, infiltrated plaque involving the left inguinal–scrotal region. (**b**) Close-up view of the lesion with sharply demarcated erythema, superficial scaling and lichenification, resistant to previous topical therapy.

**Figure 2:**
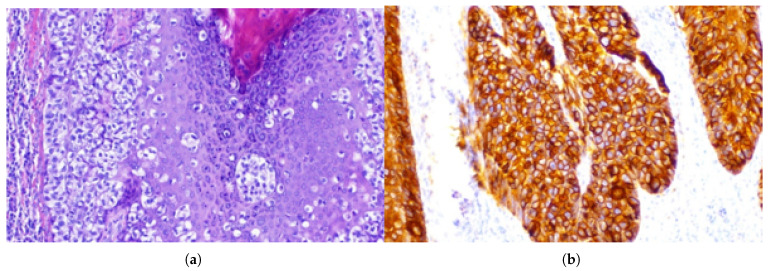
Histopathological features of extramammary Paget disease (Case I). (**a**) H&E staining showing extensive intraepidermal involvement with basilar and pagetoid spread of Paget cells in single units and small nests. (**b**) Pan-cytokeratin (AE1/AE3) immunostaining highlighting Paget cells and confirming epithelial differentiation.

**Figure 3:**
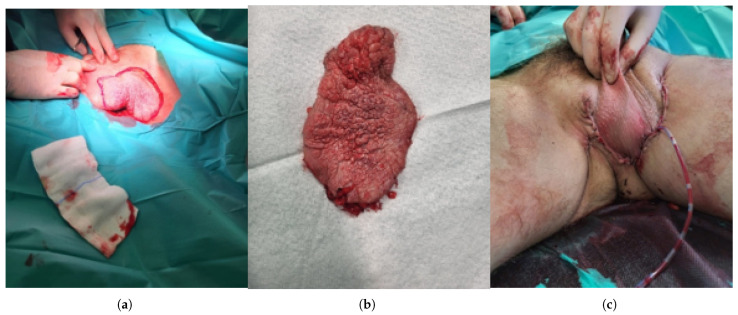
Surgical management of penoscrotal extramammary Paget disease. (**a**) Intraoperative view with clearly marked surgical margins prior to excision of the diseased scrotal and inguinal skin. (**b**) Excised specimen demonstrating an irregular, erythematous, verrucous Paget lesion corresponding to the clinically involved area. (**c**) Immediate postoperative appearance after partial scrotal and inguinal skin excision with reconstruction and placement of a suction drain.

A 60-year-old man was admitted to the Department of Urology because of lesions on the scrotal skin and in the left groin (Figure 1). The patient had previously received topical treatment in a dermatology outpatient clinic for suspected dermatitis, without clinical improvement. Subsequent histopathological examination of a biopsy specimen revealed extramammary Paget disease (EMPD) (Figure 2). His chief complaints included pruritic, dry, erythematous lesions on the scrotal and left inguinal skin, accompanied by intermittent pain in the left groin. He reported that these symptoms had been present for approximately 18 months. At admission, the patient’s weight was 87 kilograms (kg), height 182 centimeters (cm) and body mass index (BMI) 27.2 kg/m^2^. Ultrasound examination of the inguinal region revealed no enlarged lymph nodes. Comorbidities included prostate cancer (Gleason score 4 + 3, N0) treated laparoscopically and stress urinary incontinence. A partial scrotal resection was performed. An incision was made in the scrotal skin and the lateral aspect of the left thigh within macroscopically healthy margins. The diseased skin and subcutaneous tissue were dissected, and reconstruction of the scrotal and left inguinal skin was carried out (Figure 3). Histopathological examination of the excised specimen confirmed EMPD. CK7 remains the most consistently expressed and diagnostically useful cytokeratin in EMPD, whereas pan-cytokeratin markers used in our case such as AE1/AE3 primarily confirm epithelial origin. Proliferation of atypical Paget cells was present in the hyperplastic, acanthotic and papillomatous epidermis as well as in individual hair follicles. No stromal invasion was observed. The lesion was completely excised with a histological margin of 3 mm (Figure 3). Four days after partial resection of the scrotal and inguinal skin and subcutaneous tissue, 18F-FDG PET–CT was performed to exclude residual or metastatic disease. Imaging was obtained 60 min after tracer administration, from the skull base to the feet. Within the resolution limits of the method, no hypermetabolic proliferative process was identified. A few metabolically active but non-enlarged lymph nodes were noted posterior to the submandibular glands and on the left side near the carotid sheath, most likely representing reactive changes. In the left groin, moderately metabolically active lymph nodes were also interpreted as reactive/inflammatory. Minimal metabolic activity in the skin to the left of the scrotum was considered non-proliferative and recommended for local clinical follow up.

## 6. Case Presentation II: Invasive Penoscrotal EMPD Treated with Surgery, PDT and Topical Imiquimod

**Figure 4:**
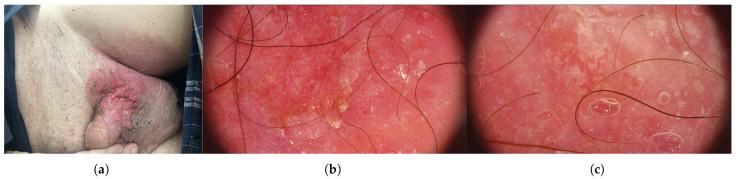
Preoperative clinical and dermoscopic presentation of penoscrotal extramammary Paget disease. (**a**) Clinical photograph showing an erythematous, infiltrated, exophytic plaque involving the left scrotal and inguinal region. (**b**,**c**) Dermoscopic images of the lesion demonstrating milky-red areas, whitish scaling and polymorphous dotted and glomerular vessels on a pink background, consistent with extramammary Paget disease.

**Figure 5:**
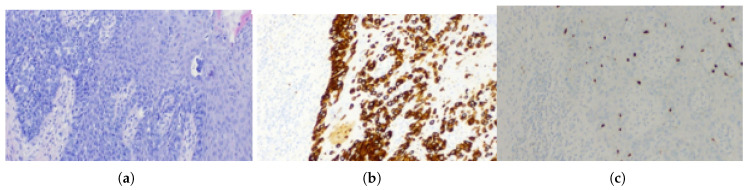
Histopathological characteristics of extramammary Paget disease. (**a**) Tissue fragment showing Paget carcinoma cells concentrated within the basal layer and dispersing into the upper portions of the epidermis. (**b**) Typical expression of cytokeratin 7 (CK7) in Paget cells. (**c**) SOX10 immunostaining: Paget cells are negative; scattered SOX10-positive background melanocytes serve as an internal positive control, supporting exclusion of melanoma.

**Figure 6:**
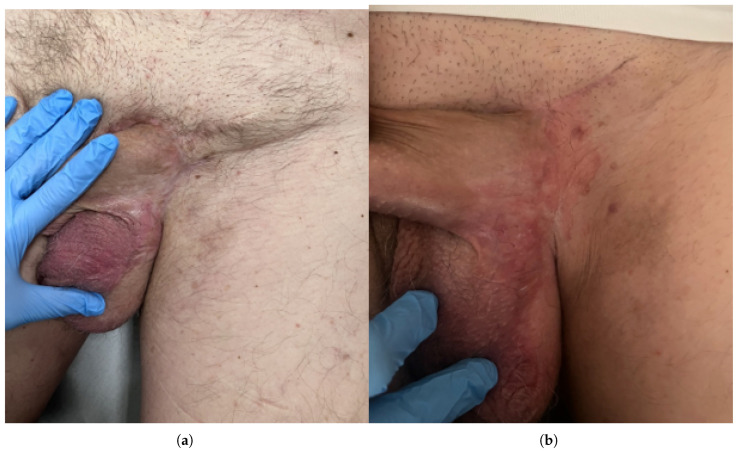
Postoperative clinical appearance after surgical treatment of penoscrotal extramammary Paget disease. (**a**) Early postoperative view showing a well-healed inguinal–scrotal scar with mild residual erythema and good contour of the reconstructed scrotal skin. (**b**) Subsequent follow up demonstrating further fading of erythema and stable postoperative result without clinical signs of local recurrence.

**Figure 7:**
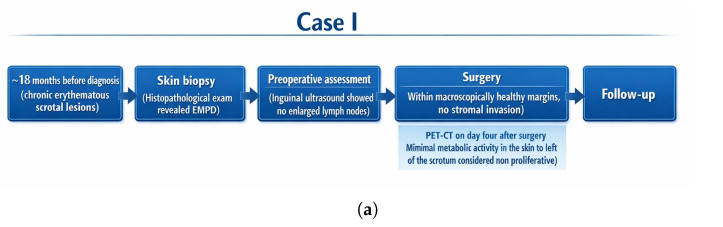

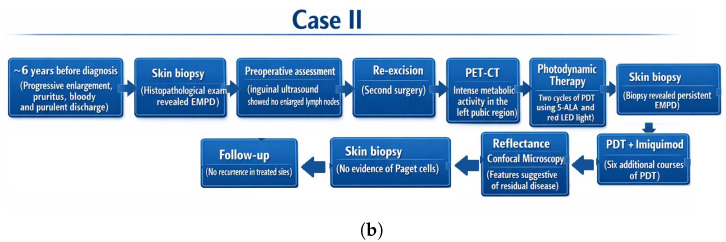
Timeline summarizing the diagnostic work-up, treatment interventions, and follow-up in the two presented cases of penoscrotal EMPD. (**a**) Case I: diagnostic biopsy confirming EMPD; partial scrotal/inguinal excision with reconstruction (no dermal invasion; negative margins); early postoperative 18F-FDG PET–CT showing no evidence of residual or metastatic disease; clinical surveillance. (**b**) Case II: diagnostic biopsy confirming EMPD; primary surgical excision (microinvasion; focally positive margin) followed by re-excision after wound healing; postoperative PET–CT with activity in the operative field (postoperative change vs. residual disease); photodynamic therapy (PDT) with 5-aminolevulinic acid guided by reflectance confocal microscopy (RCM) assessment; additional PDT sessions and topical imiquimod; serial biopsies negative for Paget cells; no clinical recurrence at last follow-up.

A 58-year-old man was admitted to the Department of Urology because of lesions on the scrotal skin and in the left groin that had been present for six years. The lesions had previously been treated topically for suspected neurodermatitis, without effect. The patient reported progressive enlargement of the lesion, pruritus and intermittent discharge of bloody and purulent material (Figure 4). Histopathological examination of a skin biopsy revealed EMPD. At admission, the patient’s weight was 84 kg, height 180 cm and BMI 25.9 kg/m^2^. Ultrasound examination of the inguinal region showed no enlarged lymph nodes. A large nodular lesion was surgically excised; it partially involved the skin at the left penile base, extended extensively to the scrotal skin towards the groin and partially involved the skin of the mons pubis. Histopathological examination of the excised specimen confirmed EMPD (Figure 5). On the skin surface there was an irregular, verrucous tumor measuring 7.7 × 6.7 cm, located 0.5 cm from the nearest surgical margin. The tumor showed superficial infiltration into subcutaneous adipose tissue. Intraepidermal carcinoma extensively involved the epidermis and hair follicles, with focal microinvasion into the stroma confirmed by AE1/3 staining. Immunophenotyping revealed the following profile: CK7+, CK5/6−, AE1/3+, SOX10− (tumor cells; scattered SOX10-positive background melanocytes served as an internal positive control) SOX10−, mucicarmine−, GATA3+, mammaglobin+/− and CDX2−. The surgical excision was assessed as focally incomplete (Figure 6). A second excision was performed three months later after complete postoperative wound healing and multidisciplinary review of the histopathology demonstrating focally positive margins. A PET–CT scan obtained after the second surgery demonstrated intense metabolic activity in the skin and subcutaneous tissue of the left pubic region, extending laterally and superiorly from the scrotum and above and to the left of the penis. However, because of the recent surgical intervention, it remained unclear whether these findings represented purely postoperative changes or overlapped with residual neoplastic disease. The patient subsequently underwent two cycles of PDT using 5-aminolevulinic acid and red LED light at 633 nm. Biopsy after the first PDT cycle revealed persistent EMPD; therefore, a second PDT series was initiated and was well-tolerated. A biopsy from newly developed lesions in the right groin demonstrated only acanthotic epidermis with mild fibrosis and no Paget cells. Reflectance confocal microscopy was used to assess lesion margins and demonstrated features suggestive of residual disease in the pubic and scrotal regions. Consequently, six additional PDT sessions were recommended, followed by topical treatment with imiquimod. During follow up, biopsies were repeatedly obtained from clinically suspicious areas in the groin and scrotum. Histopathological examination consistently showed acanthotically thickened epidermis with mild superficial dermal fibrosis and sparse perivascular lymphohistiocytic infiltrates, without evidence of Paget cells. Dermoscopy revealed few dilated vessels and seborrhoeic keratoses in the right groin but no specific features of EMPD. According to the literature, and in agreement with our findings, the immunoprofile of the lesion (CK7+, AE1/3+, GATA3+, SOX10−, CDX2−) is characteristic of primary genital EMPD and helps to exclude melanoma as well as secondary involvement by colorectal or urothelial carcinoma. At the most recent clinical evaluation, no signs of recurrence were observed at the surgically and photodynamically treated sites (Figure 7).

## 7. Discussion

Penoscrotal extramammary Paget disease (EMPD) in men is a rare and heterogeneous malignancy that poses significant diagnostic and therapeutic challenges. Its nonspecific clinical presentation, frequent delay in diagnosis and high risk of local recurrence, despite often indolent biological behavior, make early recognition, adequate staging and long-term surveillance particularly demanding for both dermatologists and urologists. In male genital EMPD, chronic eczematous lesions of the scrotum or penis are frequently misdiagnosed as inflammatory dermatoses, leading to prolonged diagnostic delay, as observed in both of our cases. EMPD of the male genital region has undergone a notable evolution in conceptual understanding. Historically, EMPD was often regarded as a manifestation of epidermotropic spread from an underlying visceral malignancy; however, accumulating clinicopathological and molecular evidence supports the existence of distinct primary cutaneous forms. Male genital EMPD represents a diagnostically and therapeutically unique subtype, characterized by frequent diagnostic delay, anatomical constraints affecting surgical management, and a clinically relevant risk of dermal invasion or associated internal malignancies. These features necessitate a tailored diagnostic and therapeutic approach distinct from EMPD at other anatomical sites. From a histopathological perspective, the exclusion of metastatic colorectal or urothelial carcinoma requires careful integration of morphological features with a targeted immunohistochemical panel. Markers commonly applied in this setting include GATA3, p63, CK20, and CK7; however, none of these markers is entirely specific when interpreted in isolation. In particular, GATA3 expression may be observed not only in urothelial carcinoma but also in normal skin and adnexal epithelial structures, which limits its diagnostic specificity. Therefore, reliable exclusion of urothelial differentiation depends on the combined interpretation of multiple markers in conjunction with architectural and cytological features. Additionally, the potential occurrence of synchronous or metachronous internal malignancies further complicates diagnostic assessment, underscoring the importance of close clinicopathological correlation and a structured, multidisciplinary approach. In this context, there is a clear need for pragmatic, real-world data that integrate modern imaging, histopathology and organ-sparing treatment options into a coherent management pathway. To our knowledge, this is among the few reports focusing specifically on male penoscrotal EMPD in a Central European setting, integrating PET–CT, RCM and PDT into a multimodal management algorithm. By combining a narrative review of the literature with two illustrative cases, we highlight how whole-body PET–CT can refine staging and surveillance, how reflectance confocal microscopy can improve margin assessment and follow-up of subclinical disease, and how photodynamic therapy and topical agents may be used as adjuncts or alternatives to extensive surgery in selected patients [52]. Treatment includes surgical and non-surgical options such as radiotherapy, chemotherapy, PDT, targeted therapy, and topical agents (5-FU, interferon, imiquimod). Radiotherapy and chemotherapy are used mainly in advanced or unresectable disease, while PDT with 5-aminolevulinic acid has shown benefit in recurrent or superficial lesions. HER2 positivity correlates with invasive behavior and poorer outcomes and enables the use of trastuzumab, which shows significant and well-tolerated responses. GATA3 is highly expressed and useful diagnostically but does not distinguish primary from secondary EMPD. AR is frequently expressed in EMPD, although reported positivity rates vary across cohorts and scoring approaches (approximately 54–90% in earlier immunohistochemical series and up to 96% in a larger semi-quantitative cohort) [53,54,55]. Importantly, higher AR expression has been associated with malignant progression (greater tumor thickness, lymph node metastasis, and higher stage) and enrichment in invasive as well as metastatic/recurrent components; however, other studies using different cut-offs did not find a significant association between AR positivity and invasion status, underscoring inter-study heterogeneity. Collectively, these data support further investigation of androgen blockade in selected AR-positive advanced EMPD. Importantly, the relationship between AR and outcomes should not be interpreted through a simplistic “male vs female incidence” lens. Sex distribution in EMPD varies substantially between cohorts, largely reflecting anatomic site and geographic setting: vulvar-dominant series are often female-predominant [56], while penoscrotal-dominant series may be male-predominant [29,53]. AR expression more plausibly reflects apocrine differentiation—normal apocrine glands are typically ER/PR-negative but consistently AR-positive—and EMPD frequently recapitulates this phenotype across both sexes [55,56]. From a clinicopathological standpoint, increasing AR expression appears to track malignant progression. In a semi-quantitative analysis, higher AR expression grade correlated with tumor thickness, lymph-node metastasis and higher stage, and AR expression was higher in invasive than non-invasive components and further elevated in metastatic/recurrent lesions [53]. Mechanistically, AR signaling may be supported by intratumoral androgen production: AR and androgen-producing enzymes (e.g., 5*α*-reductase type 1 and 17*β*-HSD5) were significantly higher in invasive versus non-invasive EMPD and correlated with proliferation/cell-cycle markers [54]. At the same time, not all cohorts have demonstrated a significant AR–invasion association [29], which may reflect methodological differences (binary positivity vs grading), sampling, and heterogeneous/patchy AR staining in intraepidermal disease compared with more diffuse staining in invasive areas. Taken together, these data provide a biologically plausible explanation for why high AR expression may be associated with poorer outcomes despite variable sex distribution across cohorts. Diagnostic evaluation relies on dermoscopy, histopathology with immunohistochemistry, and advanced imaging (RCM, PET–CT, MRI) to detect subclinical or invasive disease. Wide local excision (WLE) has high recurrence rates, whereas Mohs micrographic surgery (MMS) demonstrates substantially lower recurrence and superior margin control. Topical therapies show variable efficacy in in situ or recurrent lesions; radiotherapy provides local control in patients unfit for surgery. Systemic targeted therapy with trastuzumab, alone or with paclitaxel, offers durable responses in metastatic HER2-positive EMPD, while new HER2-directed antibody–drug conjugates such as disitamab vedotin show early promise. Immunotherapy (nivolumab, pembrolizumab, ipilimumab) has produced durable responses even in PD-L1-negative tumors, although current evidence is limited to case reports and small series. Long-term surveillance is crucial due to high recurrence risk and should include dermoscopy, repeat biopsies of suspicious lesions, advanced imaging when indicated, and periodic reassessment of biomarkers such as HER2 or AR, which may change over time. Because distinguishing primary from secondary EMPD remains difficult, all patients require evaluation for internal malignancies. Future research priorities include multicenter trials comparing treatment modalities, validation of molecular biomarkers, studies on pathogenesis including stem-cell origins, and clinical assessment of novel therapies such as next-generation HER2 inhibitors, AR blockade, CDK4 inhibitors [57], and checkpoint inhibitors. These advances may shift EMPD management toward personalized, biology-driven treatment strategies. From a broader perspective, EMPD of the male genital region illustrates the shift from a historically uniform, surgery-centered concept toward a biologically heterogeneous disease model. Early descriptions primarily emphasized local excision and the exclusion of underlying visceral malignancies, whereas contemporary studies increasingly recognize EMPD as a spectrum ranging from purely intraepidermal disease to biologically aggressive, invasion-prone tumors with distinct molecular profiles. This evolving understanding has important implications for risk stratification, treatment selection, and follow-up strategies in male genital EMPD. In this context, future progress will likely depend on integrating clinicopathological parameters with molecular and imaging-based biomarkers to refine prognostication and guide individualized management. Prospective, multicenter studies focusing specifically on male genital EMPD are needed to validate the clinical utility of biomarkers such as HER2 and AR, to define the optimal role of advanced imaging modalities, and to compare organ-sparing approaches with surgical standards. Such efforts may ultimately enable a transition from anatomy-driven to biology-driven management of this rare and challenging disease. By focusing specifically on EMPD of the male genital region, this work addresses a gap in the literature where data remain fragmented and largely extrapolated from other EMPD subtypes. Our cases and focused review aim to delineate diagnostic and therapeutic considerations unique to this anatomical site.

## 8. Conclusions

Delayed diagnosis remains a major barrier to optimal outcomes because of the non-specific clinical presentation of EMPD. Increasing awareness among dermatologists, gynecologists, urologists and oncologists is essential to reduce diagnostic delays. Educational initiatives, together with standardized histopathological and immunohistochemical protocols, can support earlier detection. Future research should integrate clinical, molecular and imaging biomarkers to refine diagnosis and personalize treatment. However, in the present cases, molecular and theranostic biomarkers were not assessed due to tissue exhaustion after routine diagnostic work-up, and therefore biomarker-related considerations should be interpreted as literature-based perspectives rather than case-specific findings. Advanced imaging techniques, particularly MRI and PET–CT, may provide valuable adjunctive information in the evaluation of extramammary Paget disease. MRI is useful for assessing local disease extent and potential dermal invasion, while PET–CT may aid in the detection of regional lymph node involvement or associated internal malignancies. Although their routine use remains a matter of debate, these modalities can be especially informative in selected cases with clinically aggressive features or ambiguous histopathological findings. Studies on patient-reported outcomes and quality of life are also needed to ensure that therapeutic advances translate into meaningful benefits for patients. The two cases presented here highlight the importance of early biopsy of persistent genital dermatoses and demonstrate how multimodal treatment—combining surgery, PDT and topical imiquimod, guided by histopathology, PET–CT and RCM—can achieve durable disease control in selected patients, even in the absence of molecular theranostic profiling. By combining earlier detection, improved education and the development of novel targeted and immunotherapeutic approaches reported in the literature, future EMPD research may reduce recurrence rates and improve prognosis in this challenging disease.

## Figures and Tables

**Table 1:** Epidemiological characteristics of extramammary Paget disease by geographic region.

Author	Region	Male-to-Female Ratio	Predominant Site	No. of Cases	Median/Mean Age	Ref.
Van der Zwan et al. 2012	Europe	1:2.8	Vulvar region	871	74 years	[15]
Ghazawi et al. 2020	Japan	1.5:1	Scrotum and penis	544	72 years	[16]
Yin et al. 2021	China	2–5:1	Scrotum, penis and groin	84	65 years	[17]
Joshi et al. 2024	Asian Americans	1:1.2	Anorectum, penis and scrotum	421	66–74 years	[18]

**Table 2:** Key molecular biomarkers and therapeutic targets in extramammary Paget disease.

Biomarker/Gene	Expression/Mutation Frequency	Clinical Relevance	Ref.
CK7, CK20	All the cases of primary EMPD exhibited the immunophenotype CK7+/CK20− (15/15)	Supports adnexal and epithelial origin; useful diagnostic marker distinguishing primary EMPD	[31]
GCDFP-15	Positive in 16/20 cases of primary EMPD and 1/6 secondary EMPD	Useful for excluding secondary EMPD of colorectal or urothelial origin	[32]
CEA	CEA positivity observed in 16/19 cases	Aids differentiation from Paget-like malignancies using immunohistochemistry	[33]
HER 2	Amplification or overexpression in 15/47 cases	Indicates PI3K/AKT and MAPK pathway activation; predictive of HER2-targeted therapies	[25]
PIK3CA	Detected mutations in 9/26 cases	Promotes oncogenesis via PI3K/AKT/mTOR signaling	[24]
RAS/RAF genes	Mutations identified in 27/144 cases	Targetable oncogenic drivers with emerging therapeutic agents	[9]
TP53	Mutation present in 7/26 patients	Implicates compromised genomic integrity and impaired tumor suppression	[24]
AR (Androgen Receptor)	Frequently expressed; reported positivity varies by cohort and scoring (approximately 54–90% across earlier IHC series). In a larger semi-quantitative cohort, AR immunopositivity was found in 98/102 primary lesions ( 96%)	Higher AR expression grade has been correlated with malignant progression (tumor thickness, lymph node metastasis, and higher stage) and is increased in invasive components and further elevated in metastatic/recurrent lesions, supporting androgen blockade as a potential therapeutic strategy in selected AR-positive advanced EMPD.	[29]
Tumor Mutation Burden (TMB)	High TMB (≥10 mutations/Mb) in 6/18 cases	May stratify patients likely to benefit from immunotherapy	[27]
Toker cells	Identified in 4/11 vulvectomy specimens (CK7-positive) Uniform positivity	Supports Toker cell origin hypothesis in vulvar EMPD	[20]
GATA 3	Uniform positivity across 71 primary genital EMPD cases	Supports apocrine lineage and adnexal differentiation	[14]

**Table 3:** Summary of treatment modalities and reported outcomes in extramammary Paget disease.

Treatment Modality	Study Type	Setting/Patient Group	Reported Outcomes	Comments/Limitations	Ref.
Wide Local Excision (WLE)	Systematic review and meta-analysis	Localized, resectable EMPD in adult patients (>18 years) with follow-up data	Recurrence rates ranging from 20–70%	High recurrence due to subclinical extension; surgical margins often inadequate	[43]
Mohs Micrographic Surgery (MMS)/CCPDMA	Systematic review and comparative studies	Primary, recurrent, or ill-defined lesions	Significantly lower recurrence rates than with WLE	Technically demanding; limited availability and time-consuming	[44]
Photodynamic Therapy (PDT)	Review	Superficial or inoperable lesions	Improved local control; lower recurrence when combined with surgery or topical therapy	Limited efficacy in invasive disease; adjunctive rather than curative treatment	[45]
Topical Imiquimod/5-Fluorouracil (5-FU)	Case report	Superficial or inoperable lesions	Partial and complete responses reported in selected cases	High recurrence rates; limited durability; requires patient compliance	[46]
Systemic Chemotherapy (taxanes, platinum agents, 5-FU)	Retrospective review	Advanced or metastatic EMPD	Transient responses and modest symptom relief; limited progression-free survival benefit	Significant toxicity; largely palliative and non-curative	[47]
HER2-targeted Therapy (Trastuzumab ± Paclitaxel)	Case report	HER2-positive advanced or metastatic EMPD	Durable and favorable therapeutic responses	Restricted to HER2-positive tumors; resistance may develop	[49]
Antibody–Drug Conjugates	Review	HER2-positive advanced or metastatic EMPD	Median progression free survival rate 5.5 months; Median overall survival: 21.9 months	Limited EMPD-specific data; requires further validation in clinical trials	[22]
Immunotherapy-Pembrolizumab	Case report	Patient with high tumor mutational burden	Temporary response; lack of sustained disease control	Efficacy limited to biomarker-selected patients; insufficient evidence	[50]
Radiotherapy	Case report	Unresectable, recurrent, or metastatic EMPD	Useful alternative therapy, particularly in elderly or unfit surgical candidates	Primarily palliative; risk of treatment-related toxicity	[51]

## Data Availability

No new data were created or analyzed in this study.

## Abbreviations

The following abbreviations are used in this manuscript:

EMPD	Extramammary Paget disease
BMI	Body mass index
CK7, CK20	Cytokeratin 7, cytokeratin 20
GCDFP-15	Gross cystic disease fluid protein-15
CEA	Carcinoembryonic antigen
HER2	Human epidermal growth factor receptor 2 (ERBB2)
PIK3CA	Phosphatidylinositol-4,5-bisphosphate 3-kinase catalytic subunit alpha
RAS, RAF	Rat sarcoma viral oncogene homolog, rapidly accelerated fibrosarcoma kinase
TP53	Tumor protein p53
AR	Androgen receptor
PD-1	Programmed cell death protein 1
PD-L1	Programmed death-ligand 1
TMB	Tumor mutational burden
PET–CT	Positron emission tomography–computed tomography
MRI	Magnetic resonance imaging
RCM	Reflectance confocal microscopy
SLNB	Sentinel lymph node biopsy
WLE	Wide local excision
MMS	Mohs micrographic surgery
CCPDMA	Complete circumferential peripheral and deep margin assessment
PDT	Photodynamic therapy
5-FU	5-fluorouracil
ADC	Antibody–drug conjugate
LED	Light-emitting diode
